# Calcium-Lignosulfonate-Filled Rubber Compounds Based on NBR with Enhanced Physical–Mechanical Characteristics

**DOI:** 10.3390/polym14245356

**Published:** 2022-12-07

**Authors:** Ján Kruželák, Klaudia Hložeková, Andrea Kvasničáková, Michaela Džuganová, Ján Hronkovič, Jozef Preťo, Ivan Hudec

**Affiliations:** 1 Department of Plastics, Rubber and Fibres, Faculty of Chemical and Food Technology, Slovak University of Technology in Bratislava, Radlinského 9, 812 37 Bratislava, Slovakia; 2 VIPO a.s., Gen. Svobodu 1069/4, 958 01 Partizánske, Slovakia

**Keywords:** rubber, lignosulfonate, glycerine, curing, morphology, tensile strength

## Abstract

Calcium lignosulfonate in the amount 30 phr was incorporated into rubber compounds based on pure NBR and an NBR carbon black batch, in which the content of carbon black was 25 phr. Glycerine, as a cheap and environmentally friendly plasticizer, was applied into both types of rubber formulations in a concentration scale ranging from 5 to 20 phr. For the cross-linking of rubber compounds, a sulfur-based curing system was used. The work was aimed at the investigation of glycerine content on the curing process and rheological properties of rubber compounds, cross-link density, morphology and physical–mechanical properties of vulcanizates. The results show that glycerine influences the shapes of curing isotherms and results in a significant decrease between the maximum and minimum torque. This points to the strong plasticizing effect of glycerine on rubber compounds, which was also confirmed from rheological measurements. The application of glycerine resulted in better homogeneity of the rubber compounds and in the better dispersion and distribution of lignosulfonate within the rubber matrix, which was subsequently reflected in the significant improvement of tensile characteristics of vulcanizates. A higher cross-link density as well as better physical–mechanical properties were exhibited by the vulcanizates based on the carbon black batch due to the presence of a reinforcing filler.

## 1. Introduction

The word lignin is derived from the Latin term lignum, which means wood [1]. Following cellulose, it belongs to the second-most-spread renewable natural polymer in the world. This aromatic biopolymer originates from the biosynthesis of the plants by the radical polymerization of three phenylpropane units: p-hydroxyphenyl (H), syringyl (S) and guaiacyl (G), which are formed from the following lignin precursors: para-coumaryl alcohol, sinapyl alcohol and coniferyl alcohol [2,3]. Those units are interconnected by a variety of C-C or ether-like C-O-C linkages to form three-dimensional highly branched polymer structures with a variety of functional groups: aliphatic and phenolic hydroxyls, carbonyl, carboxylic and methoxyl groups [4,5]. Lignin is amorphous polymer which behaves as a thermoplastic material, having glass transition temperatures Tg that can vary in a broad range depending on the isolation method, plant species, absorbed water, molecular weight and thermal history [6]. It is commonly derived from wood or crops, where it is integral part of the cell and imparts mechanical strength to wood. Thus, it can be considered as a construction material. The biggest producers of lignin are paper mills and the pulp-making industry. Lignin has many interesting properties, including a high availability, eco-friendliness, biodegradability, non-toxicity, antimicrobial and antioxidant behavior, adhesive properties, a low price, etc. [7,8,9,10,11]. Those attributes make it different from traditional fillers used in rubber industry, i.e., carbon black or silica. Despite that, only about 2% of worldwide-obtained lignin is commercially used or to produce value-added products. The rest is landfilled or used as cheap burning fuel.

Lignin is extracted from the lignocellulosic parts of wood and plants by chemical, biochemical or physical treatments. The pulping process, extraction procedure as well as the botanical source influence the lignin’s purity, structure and properties. Several extraction processes are used to obtain the so called technical lignins, and among them Kraft pulping and sulfite pulping are the most frequently used [6,12,13]. Kraft pulping is carried out in an alkali aqueous medium with a very high pH (13–14) using sodium hydroxide and sodium sulfide that accelerate the degradation of lignin. Kraft lignins contain high numbers of condensed structures and phenolic hydroxyls groups [14,15]. Most Kraft lignins are burnt for heat production, leading to their low-value utilization [12]. The sulfite process is nowadays the main source of commercially available lignins, which are commonly named lignosulfonates. This process is performed in an acid environment (pH = 1–2), using aqueous sulfur dioxide and a suitable salt-based acid. Lignosulfonates contain high amounts of sulfur in the form of sulfonate groups. Thus, they are found to be polyelectrolyte salts of the lignosulfonic acid. Anionic groups, such as sulfonate, carboxylic or hydroxyl impart water solubility. On the other hand, less polar moieties, as for instance aliphatic and aromatic, enable interactions with surfaces and interfaces [16,17]. Lignosulfonates have higher molecular weights when compared with Kraft lignins, and a broad index of polydispersity [18]. Those properties and their high availability enable lignosulfonates to be used in a wide sphere of applications, such as flocculants, surfactants, adhesives, dispersant agents, flame retardants, stabilizers, compatibilizers, binders, additives to concretes and composites, energy storage or 3D-printing applications [19,20,21,22,23,24,25,26]. In addition, their high amounts of carbon, mechanical properties, good stabilities, good viscoelastics and rheological properties make them suitable candidates as fillers for rubber compounds and composites [10,27,28,29,30,31]. Lignis and lignosulfonates are rather polar materials, and thus strong inter and intramolecular interactions are formed between macromolecules. The interactions between the macromolecules of the biopolymer are much stronger than the interactions between rubber/biopolymer, due to which the mutual miscibility and compatibility with rubber matrices are usually poor. Therefore, the incorporation of virgin lignins into rubber formulations often leads to the deterioration of composite’s mechanical properties. To improve adhesion and compatibility between both components, a combination of lignins with reinforcing fillers, or the application of various physical and/or chemical modification techniques and procedures, are required [4,32,33,34,35]. These procedures have been found to be very promising and clearly have pointed to a high application potential of lignins into rubbers. Materials with high added values have been fabricated. However, it must be noted that a lot of applied modification procedures require additional expenses and/or are time consuming. 

Acrylonitrile-butadiene rubber (NBR) is specialty type rubber with good oil resistance. The main properties of NBR depend mainly on the content of acrylonitrile structural units. The higher the amount of acrylonitrile, the higher oil resistance, the better the processability and the better the mechanical properties. However, the increase in acrylonitrile leads to the deterioration of elastic properties. It is one the cheapest specialty-type rubbers and is highly used in many industrial applications. In the current study, calcium lignosulfonate was dosed to the rubber formulations based on NBR. To improve adhesion and homogeneity between rubber and the filler, a commercially highly available, cheap and eco-friendly plasticizer, glycerine, was used. The main aim of the work was to show that an improvement of compatibility on the filler–rubber interfacial condition and a subsequent enhancement of the physical–mechanical properties can be reached by the simple addition of glycerine into rubber formulations filled with lignosulfonate, without any additional modification procedures or change in fabrication conditions.

## 2. Experimental

### 2.1. Materials

Acrylonitrile-butadiene rubber NBR, type SKN 3345 (acrylonitrile content 31–35%), was supplied from Sibur International, Russia. The NBR-based rubber batch filled with 25 phr of carbon black (CB, type N330) was compounded in Vipo, a.s. Partizánske, Slovakia. Calcium lignosulfonate, used as a biopolymer filler, with the trade name Borrement CA120 was provided by Borregaard Deutschland GmbH, Germany. The pH of the calcium lignosulfonate was 4.5. The average molecular weight of the lignosulfonate was 24,000 g·mol^−1^, with a specific surface area 3.9 m^2^·g^−1^. The elemental analysis revealed the presence of nitrogen (0.14 wt.%), carbon (46.63 wt.%), hydrogen (5.35 wt.%), sulfur (5.62 wt.%) and hydroxyl groups (1.56 wt.%) in its structure. The biopolymer filler was incorporated into rubber compounds in a constant amount—30 phr. Glycerine (86% solution), used as a plasticizer, was supplied from Sigma-aldrich, USA. Glycerine was applied into rubber formulations in a concentration scale ranging from 5 to 20 phr. For the cross-linking of the rubber compounds, a sulfur-based curing system consisting of 3 phr zinc oxide and 2 phr stearic acid (Slovlak, Košeca, Slovakia) as activators, 1.5 phr of accelerator N-cyclohexyl-2-benzothiazole sulfenamide CBS (Duslo, Šaľa, Slovakia) and 3 phr sulfur (Siarkopol, Tarnobrzeg, Poland) was used.

### 2.2. Methods

#### 2.2.1. Preparation and Curing of Rubber Compounds

In this work, two types of rubber formulations were fabricated and tested. The first type of rubber compound was based on pure NBR and a constant amount of calcium lignosulfonate—30 phr. This type of rubber compound was designated as NBR-L30. The second type of rubber compound was based on NBR carbon black batch, in which the content of carbon black was 25 phr. The amount of lignosulfonate was also kept at a constant dosage—30 phr. This type of rubber compound was designated as NBR/CB-L30. The plasticizer glycerine was applied into both types of rubber compounds in a concentration scale ranging from 5 to 20 phr. The cross-linking of rubber compounds was performed by sulfur curing system as mentioned in the section above. 

The fabrication of both series of rubber compounds proceeded in the same manner using a two-step mixing process in a laboratory kneading machine Brabender (Brabender GmbH & Co. KG, Duisburg, Germany). The temperature during compounding was set to 90 °C with a rotor speed of 55 rpm. The overall mixing process took 10 min. First, pure NBR or the NBR-based batch were plasticated for 1 min, then zinc oxide and stearic acid were added for another 1 min compounding. Subsequently, the filler was incorporated and after 2 min, glycerine was introduced. The rubber compounds were compounded for next 2 min, then taken out from the mixing chamber and cooled down in a two-roll mill. In the second step, which took 4 min at 90 °C and 55 rpm, sulfur and accelerator CBS were applied. In the final stage, the rubber compounds were homogenized and sheeted in a two-roll mill. 

The curing process of rubber compounds was performed at 170 °C and at a pressure of 15 MPa in a hydraulic press purchased from Fontijne (Fontijne, Vlaardingen, Holland), following their optimum cure time. After curing, thin sheets with dimensions 15 cm × 15 cm and thicknesses of 2 mm were obtained.

#### 2.2.2. Determination of Curing Characteristics

The curing characteristics of the rubber compounds were determined from corresponding curing isotherms, which were investigated in an oscillatory rheometer MDR 2000 (Alpha Technologies, Akron, OH, USA).

The investigated curing parameters were:

M_L_—minimum torque (dN·m)

M_H_—maximum torque (dN·m)

∆M (dN·m)—torque difference, the difference between M_H_ and M_L_

*t_c_*_90_ (min)—optimum curing time 

*t_s_*_1_ (min)—scorch time

*R_v_* (min^−1^)—curing rate index, defined as:

(1) $$R_{v}=\frac{100}{t_{c90}-t_{s1}}$$

#### 2.2.3. Determination of Cross-Link Density

The cross-link densities *ν* were determined based on the equilibrium swelling of the vulcanizates in xylene. The weighted dried samples were placed into xylene, in which they swelled with time. The weights of the samples were measured every hour until the equilibrium swelling was reached. During the measurement, the solvent diffused into rubber and disrupted almost all physical interactions within the rubber matrix. The results were the determination of the chemical cross-link densities, i.e., the concentrations of chemical cross-links within the rubber matrixes. The experiments were carried out at a laboratory temperature, and the swelling time was equal to 30 h. The Flory–Rehner equation modified by Krause [36] was then used to calculate the cross-link densities based upon the equilibrium swelling states. 

#### 2.2.4. Rheological Measurements

The dynamic viscosities of the rubber compounds were investigated using RPA 2000 (Alpha Technologies, Akron, OH, USA). The samples were analyzed under strain amplitudes from 0.15 to 700% at a constant frequency of 0.2 Hz and temperature 90 °C.

#### 2.2.5. Investigation of Physical–Mechanical Characteristics

A Zwick Roell/Z 2.5 appliance (Zwick GmbH & Co. KG, Ulm, Germany) was used to evaluate the tensile properties of the vulcanizates. The tests were performed in accordance with the valid technical standards, and the cross-head speed of the measuring device was set up to 500 mm·min^−1^. Dumbbell-shaped test samples (width 6.4 mm, length 80 mm, thickness 2 mm) were used for the measurements. The hardness was measured by using durometer and was expressed in Shore A.

#### 2.2.6. Microscopic Analysis

The surface morphologies and microstructures of the vulcanizates were observed using a scanning electron microscope JEOL JSM-7500F (Jeol Ltd., Tokyo, Japan). The samples were first cooled down in liquid nitrogen under a glass-transition temperature and then fractured into small fragments with surface areas of 3 mm × 2 mm. The fractured surfaces were covered with a thin layer of gold and put into the microscope. The source of electrons was a cold cathode UHV field emission gun, the accelerate voltage ranged from 0.1 kV to 30 kV and the resolution was 1.0 nm at 15 kV and 1.4 nm at 1 kV. The SEM images were captured by a CCD-Camera EDS (Oxford INCA X-ACT, London, UK).

## 3. Results and Discussion

### 3.1. Curing Process and Cross-Link Density

**Figure 1:**
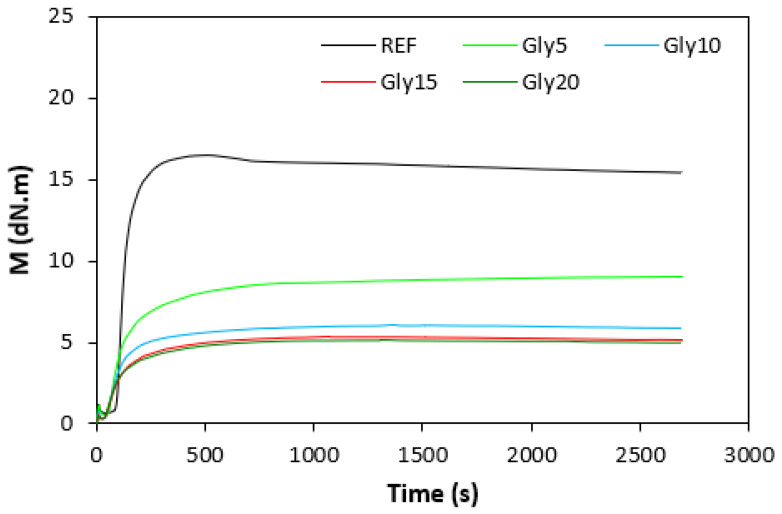
Vulcanization curves of rubber compounds NBR-L30.

**Figure 2:**
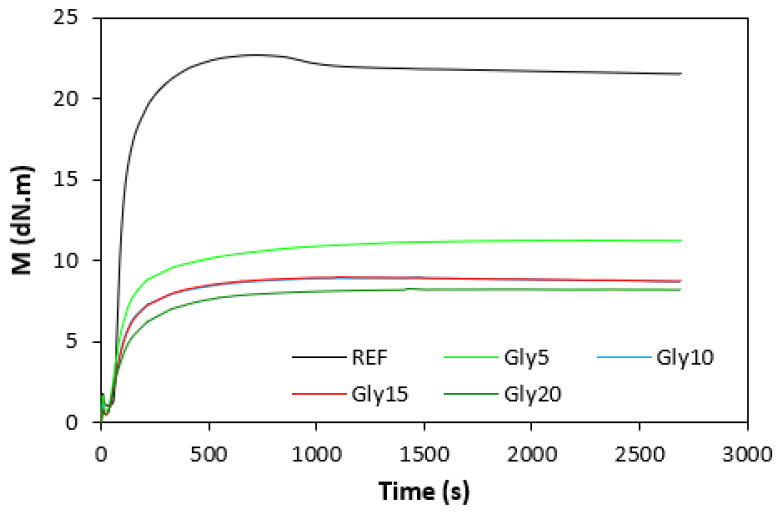
Vulcanization curves of rubber compounds NBR/CB-L30.

**Figure 3:**
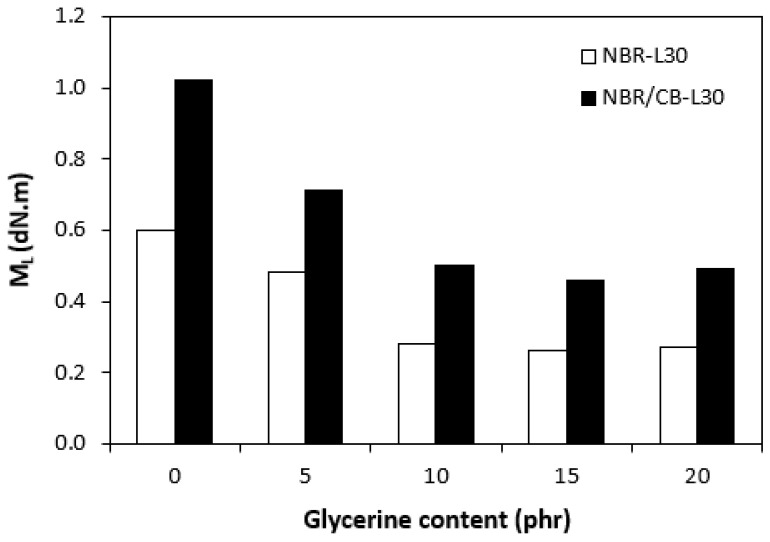
Influence of glycerine contents on minimum torques M_L_ of rubber compounds.

**Figure 4:**
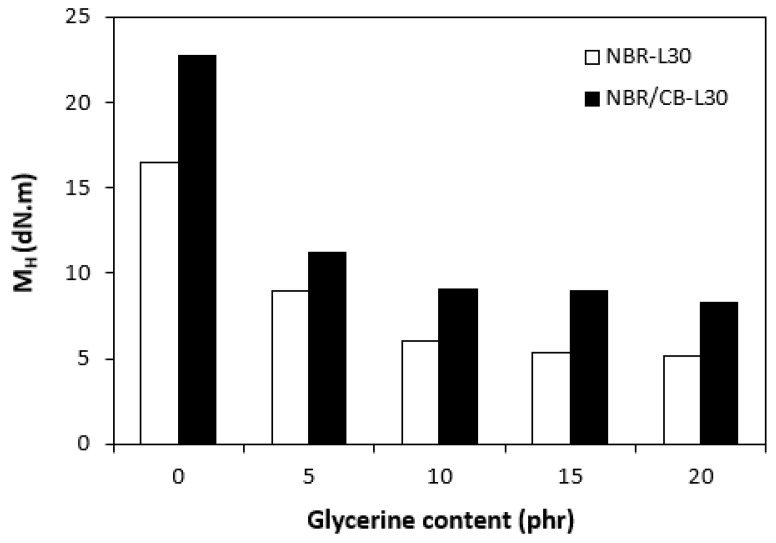
Influence of glycerine contents on maximum torques M_H_ of rubber compounds.

**Figure 5:**
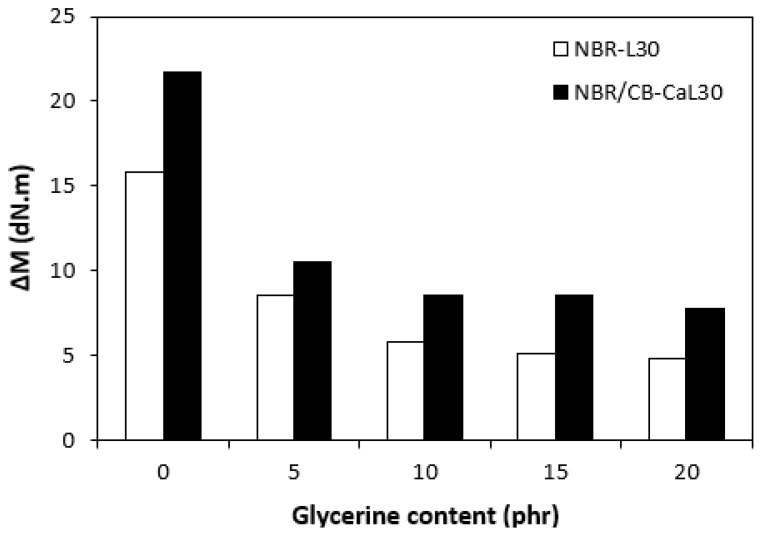
Influence of glycerine contents on torque differences ΔM of rubber compounds.

The fabricated rubber compounds were cured at 170 °C based on their optimum cure times, which were determined from the corresponding curing isotherm of each rubber compound. The curing isotherms for the rubber compounds based on pure NBR (NBR-L30) are graphically illustrated in Figure 1, while those for the rubber compounds based on NBR carbon black batch (NBR/CB-L30) are depicted in Figure 2. It becomes apparent from them that the plasticizer glycerine has significant influence on the vulcanization course for both types of rubber compound. Both the minimum torque M_L_ and the maximum torque M_H_ on the curing isotherms showed decreasing trends with increasing contents of glycerine. As shown in Figure 3 and Figure 4, higher values of both characteristics were exhibited in rubber compounds with incorporated carbon black (NBR/CB-L30). This was subsequently reflected in higher values of torque difference (ΔM = M_H_ − M_L_) for the rubber compounds based on carbon black batch (Figure 5). It also becomes clear from Figure 1, Figure 2 and Figure 5 that the higher the amount of glycerine in the rubber compounds, the lower the torque difference. This clearly points to a strong plasticizing effect of glycerine on rubber compounds. The decrease of minimum torque with glycerine loading corresponds with the decrease of the rubber compound’s viscosity before the curing process started, while the decrease of maximum torque relates to the decrease of viscosity of the cured rubber compounds. It becomes clearly apparent that glycerine acts as a plasticizer or softener for rubber compounds. Its small molecules enter the intermolecular space and disrupt inter and intramolecular physical forces between macromolecules. This leads to the increase of the rubber chain’s mobility and the reduction of the internal friction and viscosity of rubber compounds.

**Figure 6:**
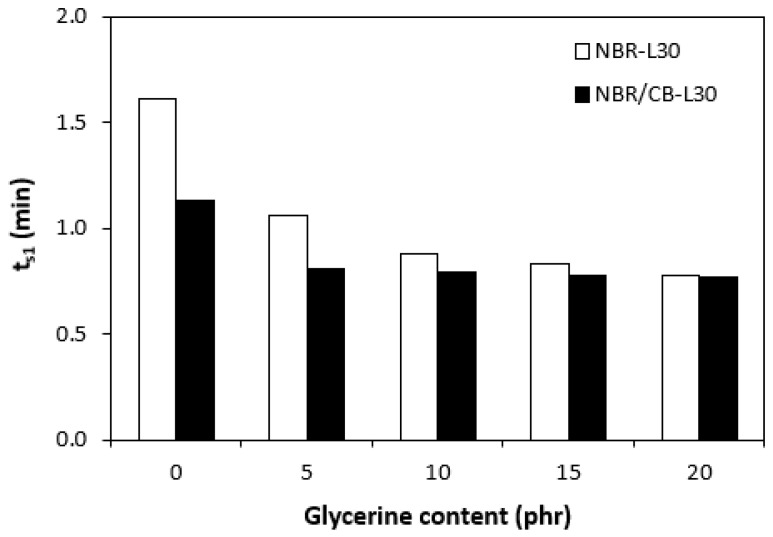
Influence of glycerine contents on scorch times t_s1_ of rubber compounds.

**Figure 7:**
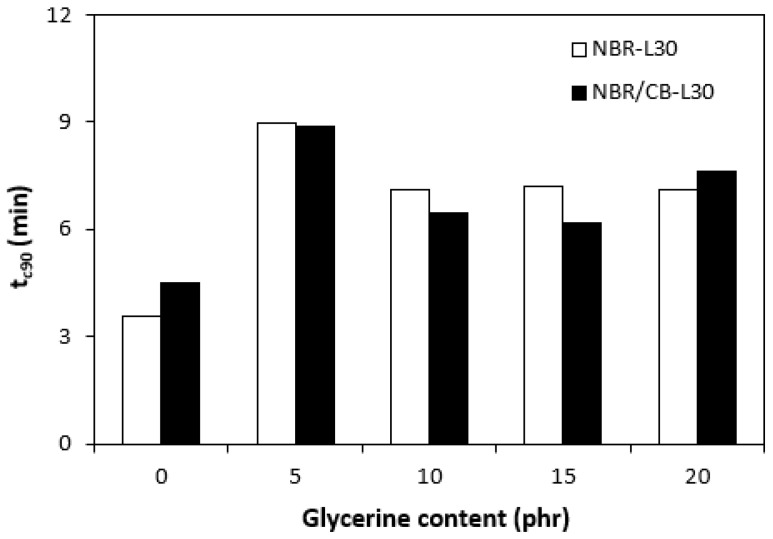
Influence of glycerine contents on optimum cure times t_c90_ of rubber compounds.

The application of glycerine caused the decrease in scorch time t_s1_ (Figure 6). Slightly higher scorch times were found to exist in the rubber compounds NBR-L30. The difference between the scorch times for both types of rubber compound became less visible with increasing contents of glycerine. The lower t_s1_ of the rubber compounds based on carbon black batch (NBR/CB-L30) can be attributed to the presence of carbon black, which enhances heat transfer within rubber compounds and thus causes them to be heated up to the curing temperature faster. On the other hand, when compared with the reference samples without glycerine, both types of rubber compounds containing 5 phr glycerine required roughly twice as long for their optimum curing (Figure 7). By next increasing the content of the plasticizer, the optimum cure time decreased and fluctuated in the low range of experimental values almost independently of the amount of glycerine. As seen in Figure 7, the optimum cure times for both rubber compound types seem to be very similar.

**Figure 8:**
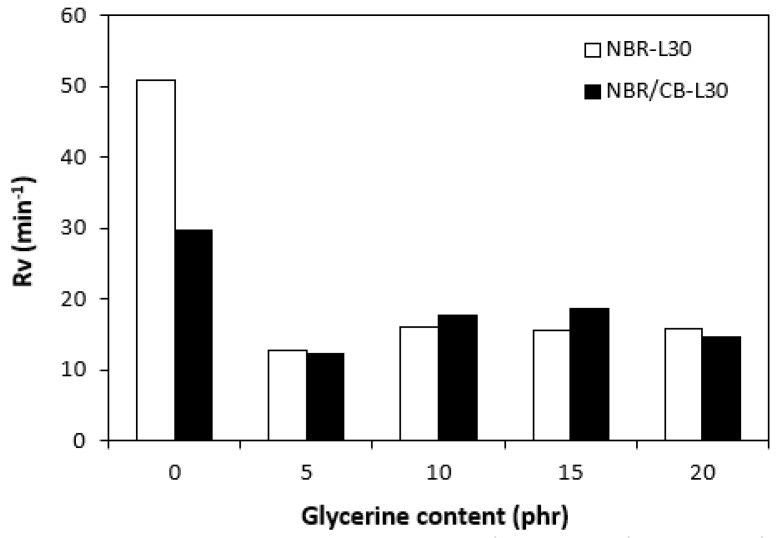
Influence of glycerine contents on curing-rate indexes R_v_ of rubber compounds.

**Figure 9:**
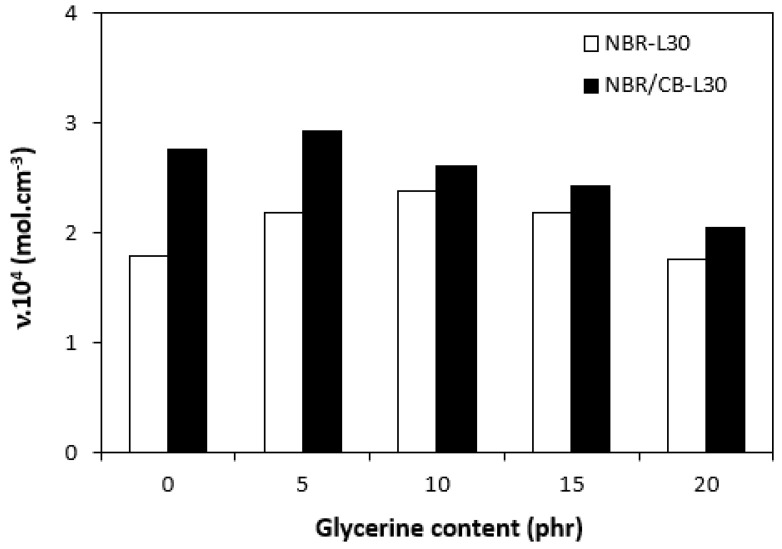
Influence of glycerine contents on cross-link densities υ of vulcanizates.

**Figure 10:**
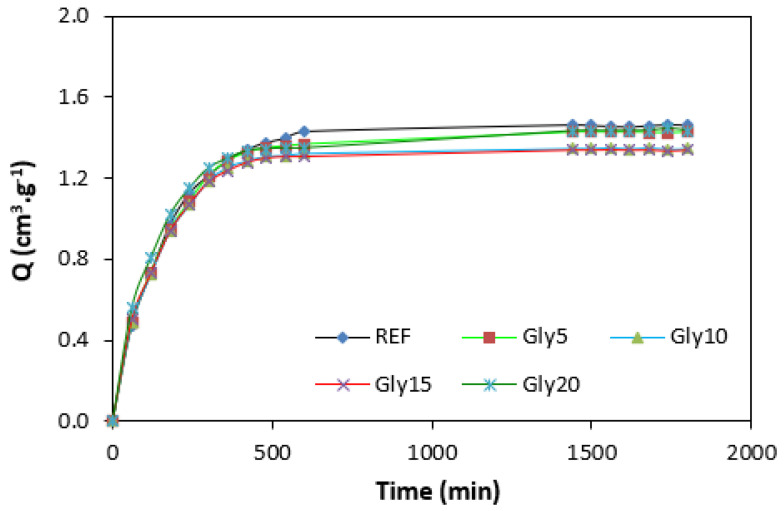
Swelling indexes of vulcanizates NBR-L30.

**Figure 11:**
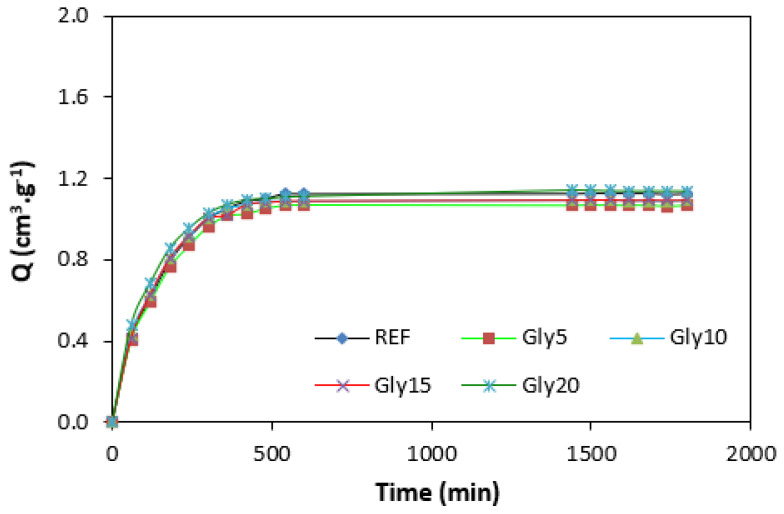
Swelling indexes of vulcanizates NBR/CB-L30.

By calculating the curing rate index R_v_ indicating the curing kinetics (Figure 8), it becomes clear that the highest curing speed exhibited the rubber compound based on pure NBR, followed by the equivalent rubber compound based on carbon black batch. The curing-rate index with glycerine-loaded rubber compounds was lower, and was found to be dependent neither on the plasticizer content, nor on the type of rubber compound. Based on the achieved results, it becomes evident that glycerine affects the course of the curing process, causes the reduction in the torque increment, and decelerates the curing speed. As already mentioned, the reduction in the torque increment can be attributed to the plasticizing effect of glycerine causing the decrease of the rubber compound’s viscosity, which was also confirmed by rheological measurement (see next chapter). The deceleration of curing kinetics can be simply explained by influencing the courses and shapes of the curing isotherms. Another possible explanation can be built on the assumption that glycerine, as a strongly polar plasticizer, can dilute or absorb curing reagents and cause them to become ineffective during curing process. The lower the amount of curing additives, the lower the curing speed. The deceleration of curing kinetics can also be caused by the acidic character of lignosulfonate. It is generally known that acidic substances have negative effects on curing kinetics and retard vulcanization. This theory could be partially supported by the decrease in the cross-link density of the vulcanizates based on carbon black batch, which upon a slight initial increase at 5 phr of glycerine, showed a decreasing trend with increasing plasticizer contents (Figure 9). The cross-link densities of the vulcanizates with the designation NBR-L30 passed over a slight maximum at 10 phr of glycerine and then dropped down. As is also obvious from Figure 9, a higher cross-link density was exhibited by the vulcanizates based on carbon black batch. This is attributed to the presence of carbon black, which forms strong physical, and even physical–chemical, interactions between carbon black particles or aggregates and rubber chains on the filler–rubber interface. Due to the strong interactions between rubber and carbon black, the rubber chains in the proximity to carbon black particles behave as a polymer in a glassy state and are insoluble in solvents [37,38]. Higher cross-link densities for the vulcanizates based on carbon black batch were then detected. The results obtained from experimental determination of the swelling index (Figure 10 and Figure 11) support the above-mentioned assumptions, showing that a lower degree of swelling was exhibited by the vulcanizates with the designation NBR/CB-L30 (Figure 11). The higher the cross-link density, the lower the free volume in the rubber matrix, and thus the lower the amount of solvent that can diffuse into the rubber compounds. It also becomes apparent that the diffusion of xylene into the rubber matrix and the swelling of the vulcanizates occurred mainly during first 6–7 h. The equilibrium swelling-degree was reached after 24 h of running the experiment. As is also shown in Figure 10 and Figure 11, no significant changes in the swelling degree were recorded regardless of the amount of glycerine.

### 3.2. Rheological Measurements

**Figure 12:**
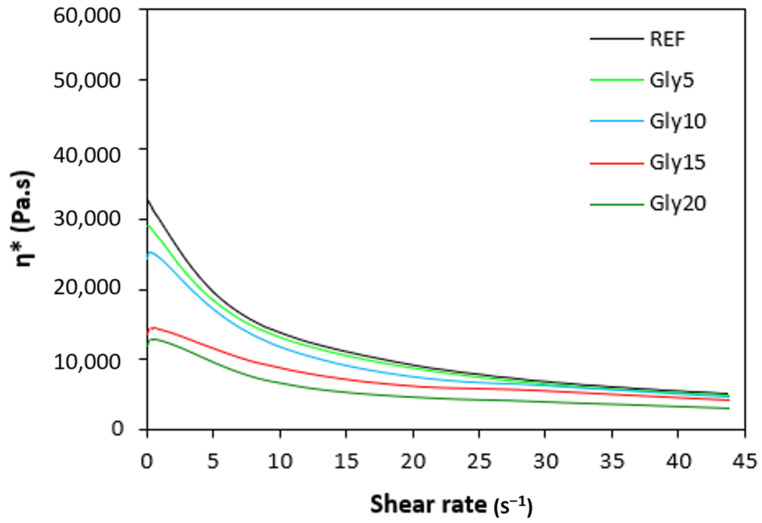
Dependence of dynamic complex viscosities η* of rubber compounds NBR-L30 on shear rate.

The dependences of the dynamic complex viscosities η* of the rubber compounds NBR-L30 on the shear rate are illustrated in Figure 12. It becomes obvious that the highest complex viscosity in the whole shear-rate range was exhibited by the reference sample without plasticizer. The application of glycerine resulted in the decrease of the complex viscosity. The lowest η* was found to come from the rubber compound with the maximum glycerine content. The differences in viscosities independently of the glycerine content were more visible at lower shear rates. With an increase in the shear rate, the viscosities of rubber compounds were found to decrease, and differences in viscosities among the rubber compounds became less visible.

**Figure 13:**
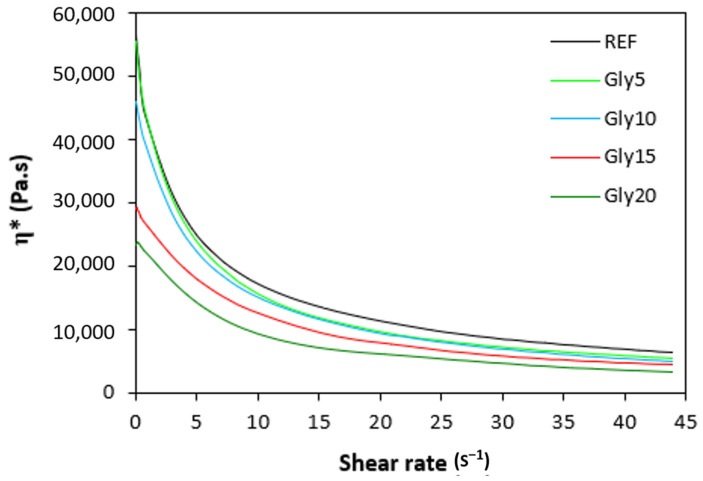
Dependence of dynamic complex viscosities η* of rubber compounds NBR/CB-L30 on shear rate.

Looking at Figure 13, one can see the similar dependences of the complex viscosities of the rubber compounds based on carbon black batch, i.e., the highest complex viscosity was found to come from the reference sample, while the lowest one was determined to come from the rubber compound with the maximum glycerine content. The higher the shear rate, the lower the viscosities, and the differences in viscosities among rubber compounds became smaller, too. When comparing the viscosities for both types of rubber formulations, it becomes clear that higher complex viscosities were demonstrated for the rubber compounds NBR/CB-L30, evidently due to the presence of carbon black. The viscosity of the filler was higher than that of rubber matrix. Second, as was already mentioned, carbon black as a reinforcing filler forms strong interfacial interactions with rubber chains, which make the rubber matrix stiffer. The rheological measurements are in line with experimental data summarized in the previous section, confirming that the application of glycerine resulted in the plasticizing of the rubber compounds and the reductions of their viscosities.

### 3.3. Physical–Mechanical Properties and Morphologies

**Figure 14:**
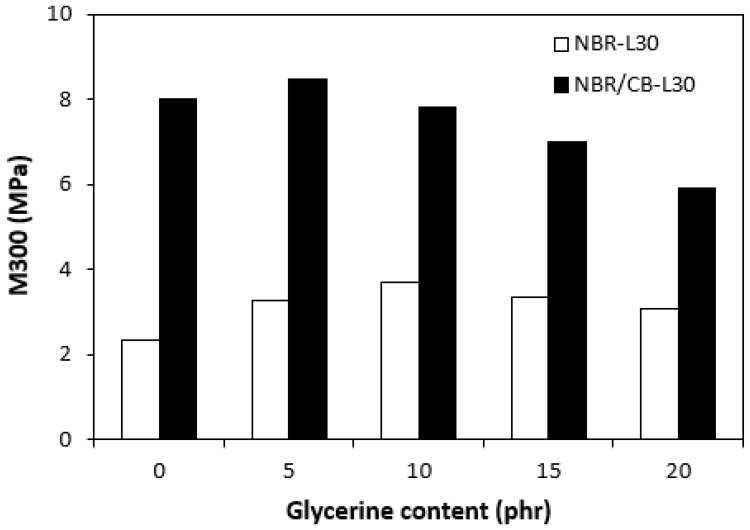
Influences of glycerine contents on modulus M300 of vulcanizates.

**Figure 15:**
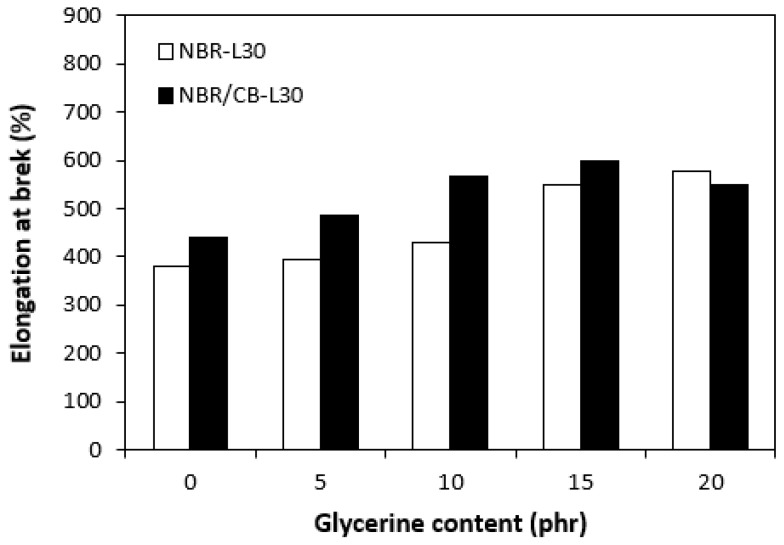
Influences of glycerine contents on elongation at breaks of vulcanizates.

**Figure 16:**
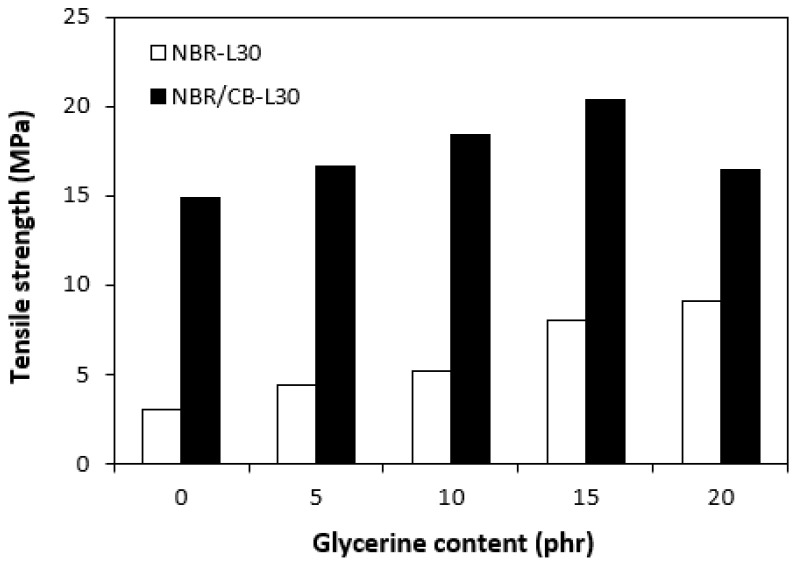
Influences of glycerine contents on tensile strengths of vulcanizates.

**Figure 17:**
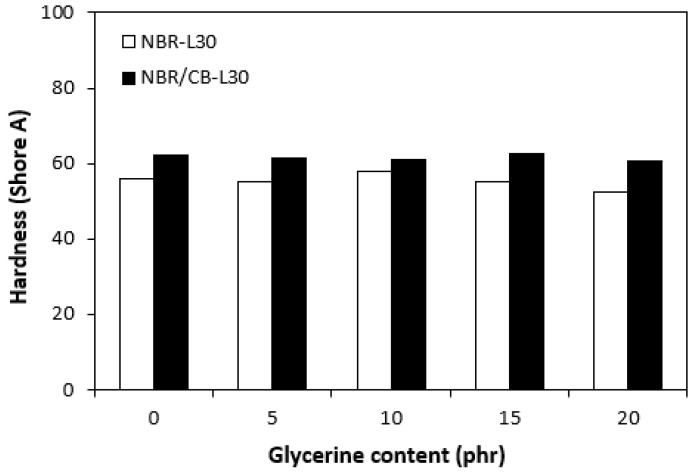
Influences of glycerine contents on hardness of vulcanizates.

The physical–mechanical properties of the vulcanizates are depicted in Figure 14, Figure 15, Figure 16 and Figure 17. The dependences of modulus M300 (Figure 14) are closely related with the dependences of the cross-link density (Figure 9). When considering the vulcanizates NBR/CB-L30, the application of 5 phr glycerine caused a slight increase in M300. Then, the modulus showed a decreasing tendency with an increasing content of glycerine. The modulus M300 of vulcanizates NBR-L30 was lower, and also followed the dependences on cross-link density, reaching a maximum at 10 phr of glycerine before dropped down.

The application of glycerine resulted in the increase of elongation at the breaks for both vulcanizate types, reaching a maximum at 15 phr glycerine for the vulcanizates NBR/CB-L30 and at 20 phr glycerine for the vulcanizates NBR-L30 (Figure 15). The increase of elongations at the breaks can be attributed to the plasticizing effect of glycerine on rubber compounds. As previously mentioned, glycerine weakens the inter and intramolecular attractive forces between macromolecules and reduces internal frictions. This leads to the increased elasticity and mobility of rubber chain segments and subsequently to the increase of elongation at the break. Similarly, the incorporation of glycerine caused the increase in tensile strength for vulcanizate types (Figure 16). The tensile strength of the vulcanizate NBR-L30 with the maximum glycerine content remarkably increased roughly threefold when compared with the reference sample (from 3 MPa for the reference up to over 9 MPa for the vulcanizate with 20 phr glycerine). The tensile strengths of the vulcanizates based on carbon black batch were much higher, owing to the presence of reinforcing carbon black, and showed an increasing trend with increasing contents of glycerine. The highest tensile strength was exhibited by the vulcanizate with 15 phr glycerine (almost 20.5 MPa), which was more than 5 MPa higher in comparison with the reference vulcanizate based on carbon black batch (15 MPa). It becomes apparent that application of glycerine leads to the enhancement of tensile characteristics for both vulcanizate types. Plasticizers are usually used to make rubber compounding easier, as they reduce the viscosity of rubber compounds and help to increase the elastic properties of vulcanizates. On the other hand, as they weaken intra and intermolecular attractive forces and physical interactions, they can also deteriorate the tensile properties of vulcanizates.

From Figure 17, it is shown that hardness of vulcanizates moved only in the low range of experimental values almost independently of the amount of glycerine. The higher hardness of the vulcanizates with designation NBR/CB-L30 is a logical reflection of the carbon black present in the rubber matrix and thus the higher overall content of fillers. The hardness of the fillers is much higher than that of the rubber. Smaller filler particles can also fill various voids and cavities within the rubber matrix, which contributes to the increase of hardness as well.

**Figure 18:**
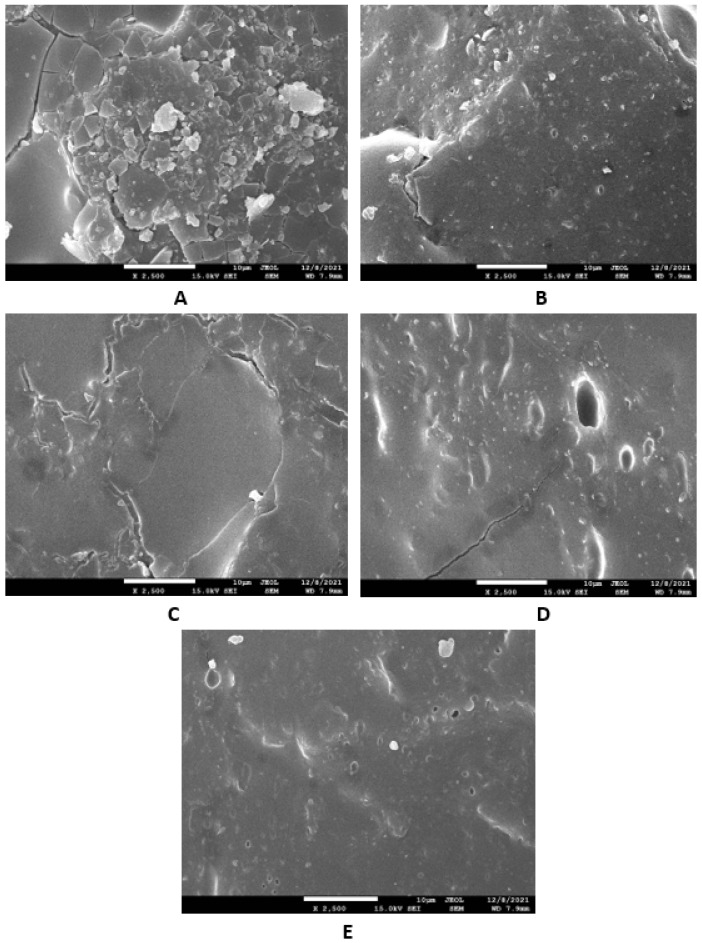
SEM images of vulcanizates NBR-L30: (**A**) reference vulcanizate without glycerol, (**B**) vulcanizate with 5 phr of glycerol, (**C**) vulcanizate with 10 phr of glycerol, (**D**) vulcanizate with 15 phr of glycerol and (**E**) vulcanizate with 20 phr of glycerol.

**Figure 19:**
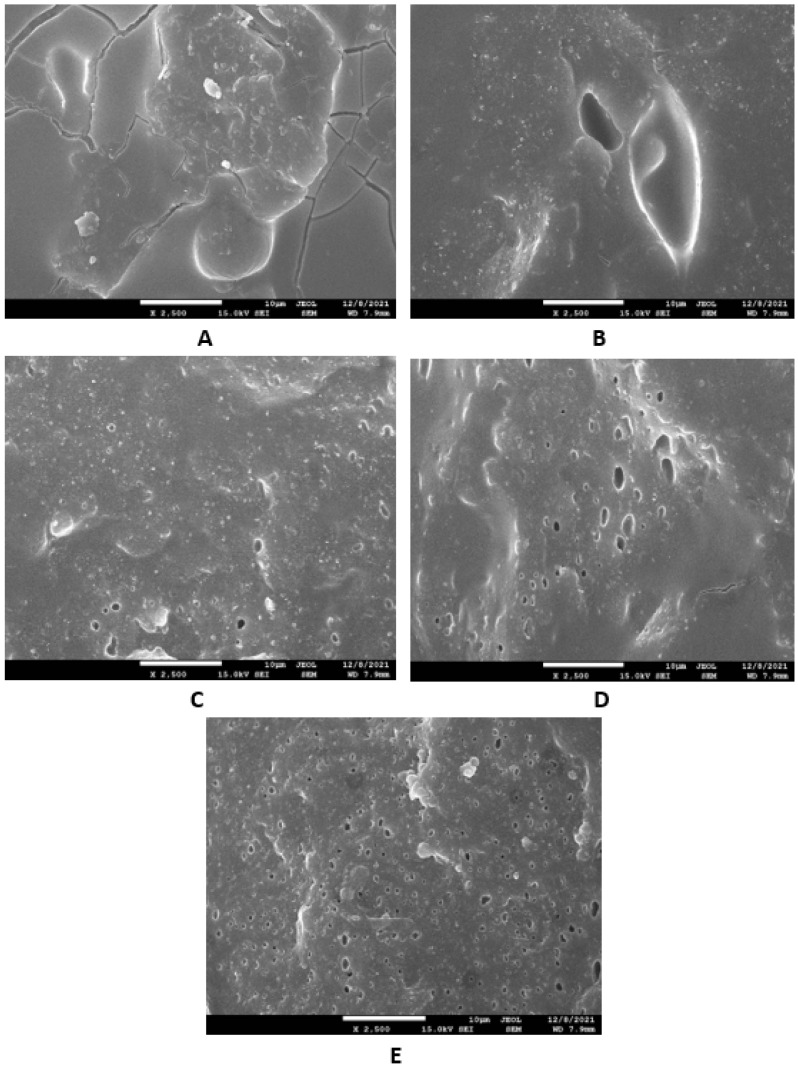
SEM images of vulcanizates NBR/CB-L30: (**A**) reference vulcanizate without glycerol, (**B**) vulcanizate with 5 phr of glycerol, (**C**) vulcanizate with 10 phr of glycerol, (**D**) vulcanizate with 15 phr of glycerol and (**E**) vulcanizate with 20 phr of glycerol.

Microscopic images unveiling the morphologies and microstructures of the surface fractures of vulcanizates NBR-L30 are presented in Figure 18. From the surface fracture of the reference, glycerine-free vulcanizate, it is shown that lignosulfonate forms lower or bigger agglomerates, which is likely caused by its insufficient dispersion and distribution within the matrix (Figure 18A). It can be stated that the homogeneity and compatibility between both components are not very good. By the introduction of 5 phr glycerine, the mutual compatibility between the rubber and the filler was substantially improved (Figure 18B). The sizes of the agglomerates were significantly reduced, and better dispersion of the filler was obtained. As seen in Figure 18B–E, the higher the amount of glycerine, the more homogenous the surface structure of vulcanizates it was possible to observe. The surface morphology of vulcanizates with designation NBR/CB-L30 is presented in Figure 19. By comparing Figure 18A and Figure 19A, it becomes apparent that the dispersion of lignosulfonate in the reference vulcanizate based on carbon black batch was much better when compared with the equivalent vulcanizate based on pure NBR. This can be attributed to the higher viscosity of the rubber compound with incorporated carbon black (Figure 12 and Figure 13). In general, the higher the viscosity of the rubber compounds, the higher the shear stress during compounding and the better the dispersion of the filler, although a higher viscosity of rubber compounds usually shifts towards a worse distribution of the filler.

**Figure 20:**
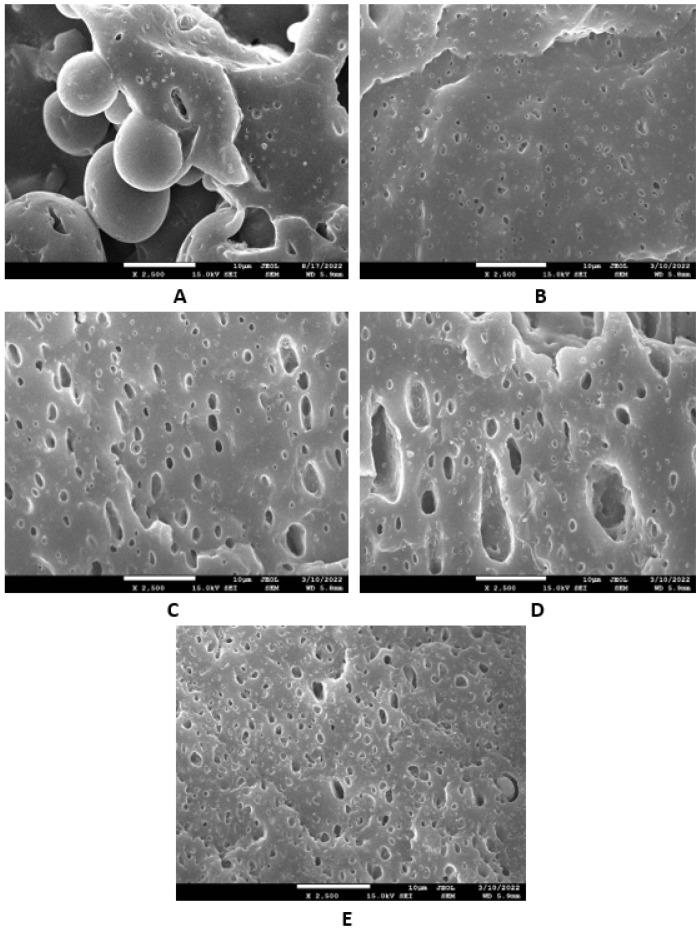
SEM images of vulcanizates NBR-L30 after washing in boiling water: (**A**) reference vulcanizate without glycerol, (**B**) vulcanizate with 5 phr of glycerol, (**C**) vulcanizate with 10 phr of glycerol, (**D**) vulcanizate with 15 phr of glycerol and (**E**) vulcanizate with 20 phr of glycerol.

**Figure 21:**
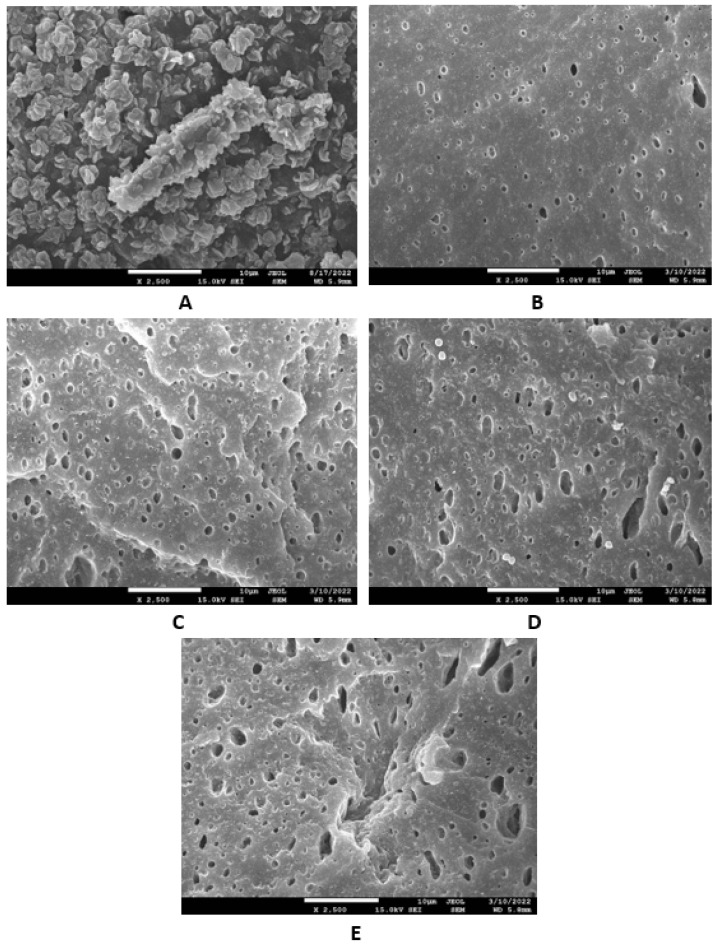
SEM images of vulcanizates NBR/CB-L30 after washing in boiling water: (**A**) reference vulcanizate without glycerol, (**B**) vulcanizate with 5 phr of glycerol, (**C**) vulcanizate with 10 phr of glycerol, (**D**) vulcanizate with 15 phr of glycerol and (**E**) vulcanizate with 20 phr of glycerol.

Based on the achieved results, it can be stated that the incorporation of glycerine resulted in the very-good dispersion and distribution of the filler within the rubber compounds with designation NBR-L30 (Figure 18B–E), as well as NBR/CB-L30 (Figure 19B–E). To deeply investigate the dispersion and distribution of the filler within the rubber matrix, the surface morphologies of vulcanizates was also studied after the vulcanizates were washed in boiling water for 2 h. As lignosulfonate is water soluble, it was extracted from the surface structure by boiling the water. SEM images of vulcanizates after washing in boiling water are presented in Figure 20 and Figure 21. It becomes obvious from them that the application of glycerine resulted in the much-higher dispersion and distribution of lignosulfonate within the rubber matrix and formed smaller domains.

Microscopic analysis also clearly revealed that the application of the plasticizer led to better homogeneity and adhesion between the lignosulfonate and the rubber matrix on the filler–rubber interface in both vulcanizate types. The surface structures became smoother and more compact, with no evident structural defects. Glycerine is hydrophilic, polar-low molecular weight plasticizer, and it is suggested that it reduces the softening point of lignosulfonate, which is also polar in nature [39]. Simultaneously, it acts as a plasticizer for rubber compounds and leads to the lowering of their viscosities, as is clearly evident from Figure 12 and Figure 13. The effects of better distribution and dispersion of lignosulfonate within the rubber matrix by the application of glycerine can be attributed to the softening effect of lignosulfonate and the adjustment of its viscosity closer to that of the viscosity of the rubber during compounding. Glycerine is also proposed to act as rubber–filler interfacial compatibilizer contributing to the good adhesion and compatibility between the two components. As mentioned in the introduction, lignis or lignosulfonates are generally inactive fillers and usually deteriorate the physical–mechanical properties of vulcanizates. This is mainly due to their poor dispersion and compatibility with rubber matrices, as well as being due to the fact that they form agglomerates and hard domains. In general, agglomerates and domains with high rigidities act as stress concentrators under the influence of external stress, and thus they deteriorate the mechanical properties of vulcanizates. Glycerine reduces the viscosity of biopolymer filler and makes it softer. Lignosulfonate can thus form softer domains with more flexible structures, which becomes clearly obvious from Figure 20 and Figure 21. The domains with lower stiffnesses can deform more easily upon external deformation, similarly to the network formed from particles of reinforcing fillers [40,41]. The above-mentioned aspects subsequently resulted in the improvement of the tensile strength of the vulcanizates.

## 4. Conclusions

Two types of rubber compounds, one based on pure NBR and one NBR-based carbon black batch with incorporated calcium lignosulfonate, were plasticized with glycerine. The effect of plasticizer on the cross-linking process, morphology, rheology and physical–mechanical properties of vulcanizates was evaluated. 

The presence of glycerine in the rubber compounds influenced the courses of curing isotherms and decelerated the curing process. The effect of glycerine on cross-link density seems to not be significant. Higher cross-link densities were found within the vulcanizates based on carbon black batch. The rubber compounds based on carbon black batch exhibited higher viscosities, though the plasticizing effect of glycerine led to the decrease of viscosity for both types of rubber compounds. Glycerine had a softening effect on lignosulfonate, which contributed to the better dispersion and distribution of the biopolymer filler within the rubber matrix. Good adhesion and compatibility between the rubber and the filler on the filler–rubber interface, along with the ability of the softened lignosulfonate to make softer domains with lower stiffnesses, resulted in the improvements of the tensile characteristics of both vulcanizate types. The tensile strength and elongation at the break showed increasing trends with increasing amounts of glycerine in rubber formulations. However, the influence of plasticizer on the modulus M300 and hardness was less visible. Better physical–mechanical characteristics were exhibited by the vulcanizates based on rubber batch due to the presence of the reinforcing carbon black. The incorporation of the cheap and eco-friendly plasticizer contributed to the improvement of the physical–mechanical properties of the lignosulfonate-filled rubber compounds, which clearly points to the high application potential of biopolymer fillers into rubber compounds. This leads to the reduction of the costs of the final rubber products, as well as emphasizing more-and-more-pronounced ecological aspects.

## Data Availability

The data presented in this study are available on request from the corresponding author.
